# Genome-Wide Analysis Reveals Loci Encoding Anti-Macrophage Factors in the Human Pathogen *Burkholderia pseudomallei* K96243

**DOI:** 10.1371/journal.pone.0015693

**Published:** 2010-12-22

**Authors:** Andrea J. Dowling, Paul A. Wilkinson, Matthew T. G. Holden, Michael A. Quail, Stephen D. Bentley, Julia Reger, Nicholas R. Waterfield, Richard W. Titball, Richard H. ffrench-Constant

**Affiliations:** 1 Biosciences, University of Exeter, Penryn, United Kingdom; 2 The Wellcome Trust Sanger Institute, Hinxton, Cambridge, United Kingdom; 3 Department of Biology and Biochemistry, University of Bath, Bath, United Kingdom; 4 School of Biosciences, University of Exeter, Streatham Campus, Exeter, United Kingdom; Charité-University Medicine Berlin, Germany

## Abstract

*Burkholderia pseudomallei* is an important human pathogen whose infection biology is still poorly understood. The bacterium is endemic to tropical regions, including South East Asia and Northern Australia, where it causes melioidosis, a serious disease associated with both high mortality and antibiotic resistance. *B. pseudomallei* is a Gram-negative facultative intracellular pathogen that is able to replicate in macrophages. However despite the critical nature of its interaction with macrophages, few anti-macrophage factors have been characterized to date. Here we perform a genome-wide gain of function screen of *B. pseudomallei* strain K96243 to identify loci encoding factors with anti-macrophage activity. We identify a total of 113 such loci scattered across both chromosomes, with positive gene clusters encoding transporters and secretion systems, enzymes/toxins, secondary metabolite, biofilm, adhesion and signal response related factors. Further phenotypic analysis of four of these regions shows that the encoded factors cause striking cellular phenotypes relevant to infection biology, including apoptosis, formation of actin ‘tails’ and multi-nucleation within treated macrophages. The detailed analysis of the remaining host of loci will facilitate genetic dissection of the interaction of this important pathogen with host macrophages and thus further elucidate this critical part of its infection cycle.

## Introduction

The Gram-negative bacterium *Burkholderia pseudomallei* is a serious environmental pathogen of man and the causative agent of the often fatal disease melioidosis. Disease occurs following exposure to contaminated water or soil, usually through cuts in the skin or via inhalation, but the underlying mechanisms of pathogenicity of *B. pseudomallei* to humans remain poorly understood [1]–[3]. *B. pseudomallei* is endemic to S.E. Asia and N. Australia where infections are associated with both antibiotic resistance and high mortality rates (∼50%). The ability of this pathogen to infect via inhalation has necessitated its listing as a potential bio-warfare agent [4]. The high rates of infection and subsequent patient mortality therefore make *B. pseudomallei* a high priority for research and vaccine development as no effective vaccine currently exists [3], [5]. During the establishment of successful infection *B. pseudomallei* adheres to, survives and replicates within host epithelial cells and macrophages by somehow interfering with the cellular mechanisms which would otherwise destroy them. Known bacterial factors affecting the interaction with host cells include the bacterial capsule, and effectors delivered by the type III and type VI secretion systems (T3SS and T6SS) [5]. The Bsa T3SS and its delivered effector BopE is associated both with invasion of non-phagocytic cells and also subsequent vacuolar escape [6]–[10]. Type VI secretion system-5 (T6SS-5) is specifically induced upon exposure to macrophages and also appears to play a role in intracellular survival [11]. Once inside the macrophage the pathogen induces macrophage cell fusion leading to the formation of so called Multi-Nucleated Giant Cells or MNGCs, a process key to both intracellular replication and bacterial persistence but one for which the molecular basis is obscure [12]. Once intracellular replication of the pathogen has reached a critical point the bacteria induce host cell death, again by an unknown mechanism, and subsequently escape host cells to establish secondary infections [3]. Importantly, different *Burkholderia* strains show a wide range of different interactions with human macrophages, ranging from no effect, to host cell apoptosis and capase-1-dependent lysis [3], [13]. This range of different responses to macrophages suggests that the complement of anti-macrophage virulence factors encoded by the genome of different strains may differ dramatically and may also indicate potential functional redundancy amongst such factors. Importantly, conventional genomic analysis has failed to identify homologues of known toxins in *B. pseudomallei* [14]. Thus for example whilst a cytolethal exotoxin has been identified in the culture filtrate of *B. pseudomallei* the toxin remains to identified and encoding gene characterised [15]. Here we therefore perform a simple gain of function screen in recombinant *Escherichia coli* to identify the full list of loci potentially encoding toxins, or other factors, with anti-macrophage activity.

To perform the screen we chose the strain *B. pseudomallei* K96246, a clinical isolate, whose genome, of two chromosomes, has been fully sequenced [14]. Chromosome 1 (4.07 Mb) represents 56% of the genome and contains a higher proportion of coding sequences (CDSs) than the smaller chromosome 2 (3.17 Mb). The CDSs on chromosome 1 are thought to be largely involved in housekeeping functions, such as metabolism, whereas those on chromosome 2 appear to encode accessory functions facilitating adaptation to atypical conditions, osmotic protection, secondary metabolism, iron acquisition and gene regulation [14]. There are predicted to be at least 16 horizontally acquired genomic islands located in the *B. pseudomallei* genome which often contain genes encoding hypothetical virulence factors [16].

Libraries of recombinant *E. coli* each carrying end-sequenced *B. pseudomallei* genomic fragments (fosmids or Bacterial Artificial Chromosomes (BACs)) were used to identify loci encoding factors cytotoxic to the murine macrophage cell-line J774-2. The end-sequences of multiple positive clones recovered from the screens were aligned on to the sequenced genome in order to identify and confirm the precise configuration of the loci involved [14]. Such a rapid and simple gain of function screen proves an extremely useful tool for dissecting pathogens displaying functional redundancy of multiple virulence factors and toxins. Such a multiplicity of bacterial virulence factors encoded within a single genome can frustrate attempts to dissect virulence via conventional mutagenesis. For example, targeted knock-out of the toxin Mcf1 in *Photorhabdus* bacteria does not dramatically decrease anti-insect virulence due to the remaining copy of a second toxin encoding gene Mcf2 and a host of other remaining virulence factor encoding genes that remain unaffected [17]. Such ‘functional redundancy’ can therefore potentially mask the important role of specific gene candidates if other virulence factors compensate for the expected change in the resulting single mutant phenotype. Given the wealth of potential genes encoding putative virulence factors in the different genomes of *B. pseudomallei*, here we use this gain of function screening technique to reveal over 100 loci encoding anti-macrophage factors scattered across the two different chromosomes of this bacterial genome. Such analyses will facilitate the identification and follow-up of effector proteins and small molecules that influence the potentially complex interaction between *Burkholderia* bacteria and host macrophages.

## Results

### Loci encoding anti-macrophage factors are distributed over both chromosomes

We screened genomic libraries of *B. pseudomallei* K96243 in BAC and fosmid clones for activity towards J774-2 macrophages. To identify and confirm anti-macrophage encoding loci in the *B. pseudomallei* genome the end-sequences from positive library clones (clones shown to reduce macrophage viability by >40 %) were re-assembled onto the sequenced genome. Each locus was defined by the minimum region of genetic overlap formed by a cluster of two or more positive clones. Using these criteria, a total of 59 anti-macrophage encoding loci were identified on chromosome 1 and 54 on chromosome 2. Details of the loci, the predicted open reading frames that they contain and associated BLAST matches are provided in detail in the supporting information (Table S1 and S2).

**Figure 1:**
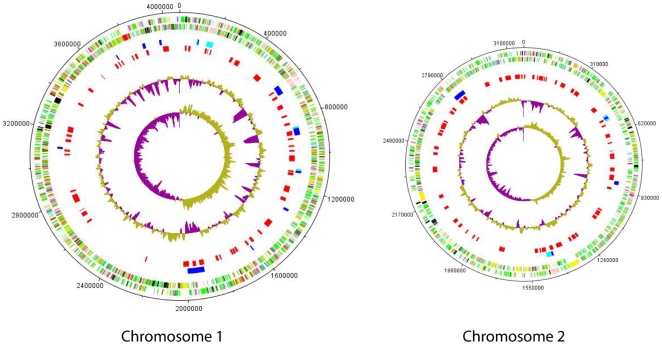
The distribution of positive loci on chromosomes 1 and 2. Outer pair of concentric circles represents both coding strands of the *B. pseudomallei* genome. The next ring in shows the location of current genomic islands (dark blue) and putative genomic islands (turquoise) and the innermost ring the location of the anti-macrophage associated loci (red). Also shown is the percentage of G+C content and the GC deviation plot (>0% =  olive, <0% =  magenta).

The screen reveals a broadly even distribution of anti-macrophage associated loci across both chromosomes with some notable exceptions (Figure 1). For example, on chromosome 1 there is a 0.6 MB region between 2,016,700 and 2,634,300 bp containing only a single region of interest. The overall density of hits is higher on chromosome 2, especially in view of its smaller size. Interestingly, target loci were found either directly within, or closely associated with, 11 out of the 16 genomic islands of strain K96243. Further, almost all of the hits are associated with regions of low G+C content (Figure 1). Both of these observations are consistent with the anti-macrophage loci being associated with genomic islands (GIs) and/or being recently horizontally transferred. A large cluster of positive loci are associated with GI 8 on chromosome 1 and GI 16 on chromosome 2. GI 8 is described as an island containing various virulence related factors including hemolysin, YadA, transport and several regulatory proteins [14]. GI 16 contains a hemagluttinin-related protein (BPSS2053) mutation of which has been shown to reduce adherence to human buccal epithelial cells [18]. However, approximately 35% of the genes contained within the *B. pseudomallei* GIs encode hypothetical proteins and their exact functions therefore remain unknown [18].

**Figure 2:**
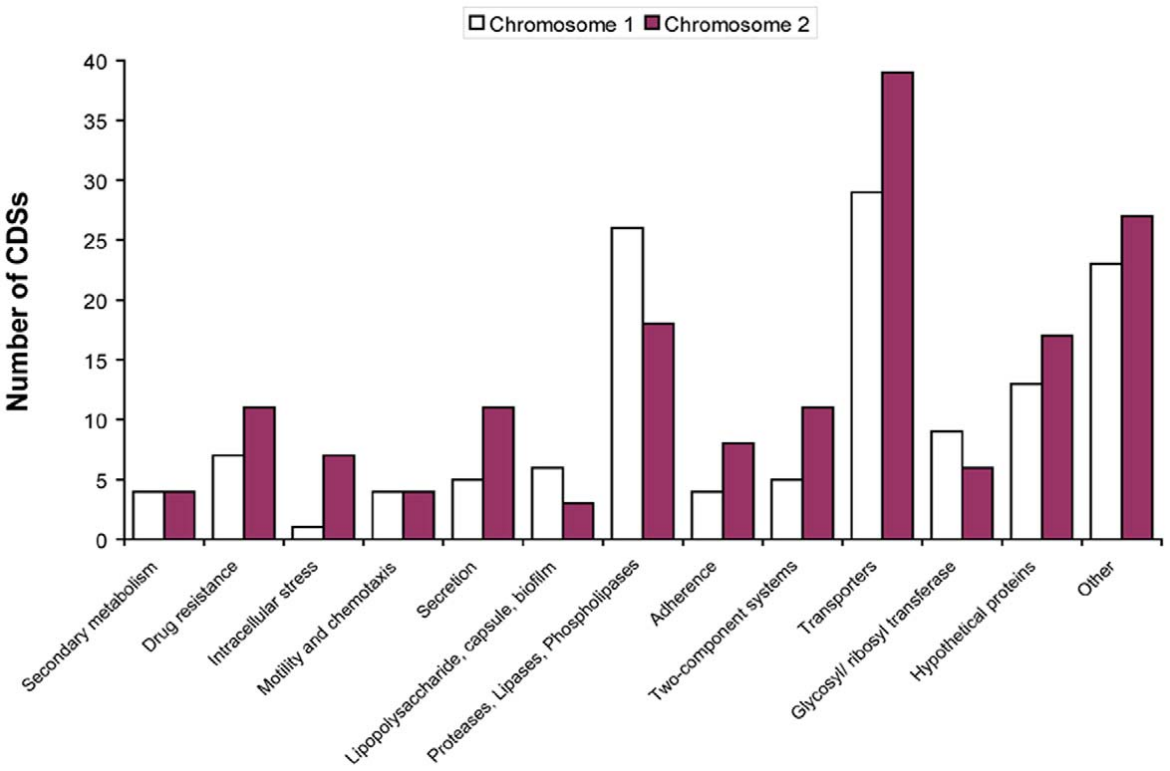
Distribution of features within positive loci on chromosome 1 and 2. Graph showing the number of CDSs for different functional classes found within anti-macrophage loci identified by the screen on both chromosomes. The majority of CDSs identified within positive loci are associated with exoprotein/ enzymic activity and transport or secretion systems.

Several broad functional classes were repeatedly predicted for the anti-macrophage loci identified (Figure 2): There are 14 predicted regions in K96243 containing putative secondary metabolite synthesis genes, 3 on chromosome 1 and 11 on chromosome 2. Six loci isolated by the screen contained clusters of genes predicted to encode non-ribosomal peptide synthetases (NRPSs) and polyketide synthases (PKSs). Genes involved in signal response and regulation (methyl-accepting chemotaxis proteins, two-component sensor systems, AraC and LysR family regulators) were also repeatedly detected. Numerous phage-related elements flank positive loci indicating that these may be mobile elements associated with insertion events, and could be responsible for the potential transferral of virulence factors between *B. pseudomallei* strains. A number of genes which could promote the survival of *B. pseudomallei* in host cells were also identified in the regions of interest including genes encoding factors responsible for resistance to drugs, heavy metals and reactive oxygen species (*sodB* and *katB*). Regions associated with anti-macrophage activity also include CDSs relating to adhesion (hemagglutinin, YD repeat proteins) and biofilm formation (lipopolysaccharide, acetolactate synthase). However the largest predicted function categories associated with the anti-macrophage loci were either toxin/enzyme related (hemolysins, proteases, phospholipases) with 44 associated CDSs within loci, or proteins involved in transport/secretion (ABC transporters, efflux transporters, and proteins involved in type III and VI secretion) with 84 related CDSs within positive loci.

Several of the anti-macrophage loci encode putative toxins. For example, BPSS1993 encodes a metalloprotease A, termed MrpA, a 47 kDa protein negatively regulated by QS molecules, a putative hemolysin with homology to *Bacillus cereus* hemolysin III (BPSS0803) and LasA elastase from *Pseudomonas aeruginosa* (BPSL0624). One locus also contains a gene (BPSS0067) encoding phospholipase – PLC-3 a putative non-hemolytic phospholipase C with an N-terminal twin-arginine translocation (Tat pathway signal sequence) which was transcriptionally up-regulated in a hamster model of infection and increases the LD_50_ of test animals dramatically [19]. Interestingly, the phospholipases PLC-1 (BPSL2403) and PLC-2 (BPSL0338) were not detected in this screen.

The genome of *B. pseudomallei* K96243 contains six gene clusters encoding putative T6SSs [11]. Whilst, again, the host *E. coli* used in the library screens were not armed with any specific type III or type VI secretion systems, several positive loci encode putative VgrG-like T6SS-related proteins. Some VgrG effector proteins have been inferred to induce host cell toxicity by ADP-ribosylation of actin, as well as performing a role in formation of the secretion machinery itself [20], [21]. *B. pseudomallei* T6SS-5 is induced upon exposure to macrophages and plays an important role in the intracellular survival of the pathogen [11]. However, a genomic clone containing the entire T6SS-5 cluster was not detected in the current study, presumably as either it did not fit entirely within one of the cloned library fragments or because other elements necessary for its production in the recombinant *E. coli* used were absent.

There are a total of 105 predicted functional ABC systems encoded within the genome of strain K96243 [22] and 22 of these were identified within our list of anti-macrophage encoding loci (14 on chromosome 1 and 8 on chromosome 2). Within this category of hits the positive ABC transporter encoding loci include two class I export systems. One region of interest detected contains the LPS biosynthetic operon BPSL2672-BPSL2688 containing the class I *wzt* ABC transporter, confirming the potential role of LPS in interaction with macrophages [23], [24] and suggesting that an active form of LPS can be correctly assembled by the host *E. coli* used for library construction. The second class I ABC system detected, BPSL3092-BPSL3094 is classified as involved in hemolysin export, however there are no identified hemolysins associated with this system in K96243 [22]. However, this ABC system neighbors two putative peptidase encoding genes whose products may be exported. Further, we note that many of the predicted proteins from positive candidate regions are thought to encode outer membrane proteins, again consistent with their potential interaction with host macrophages and their presumptive display on the outer membrane of the recombinant *E. coli* used in the screen. In summary, given that the host *E. coli* used in the screen (DH10B and EPI-300-T1^R^) lack the specific secretion machinery (e.g. T3SS or T6SS) necessary for delivery of many types of known effectors, the clusters of anti-macrophage loci uncovered here appear to be able to reconstitute toxicity in the recombinant *E. coli* either using systems still present (e.g. type 1 secretion) or by using transporters (e.g. ABC) or synthetic machinery (e.g. liposaccharide biosynthesis) also encoded within the positive clones/clusters.

### Cellular phenotypes of anti-macrophage factors

**Figure 3:**
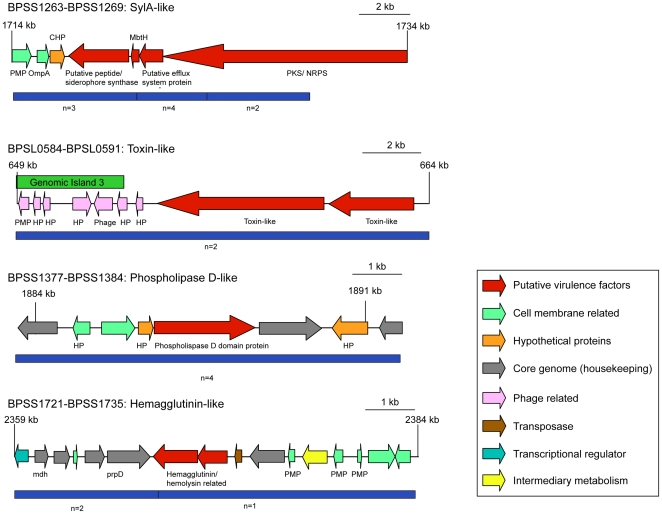
Genetic maps of four putative regions of interest. Genomic organisation of four anti-macrophage loci isolated by the screen representative of the main functional classes identified. Genes putatively involved in anti-macrophage activity are highlighted in red and roles of other genes in the region are provided in the figure legend based on the original genome annotation and BLASTX analysis. The number of overlapping positive clones isolated by the screen that identify these as regions of interest are shown in blue below the ORF maps. BPSS1263-BPSS1269 encodes NRPS/PKS genes involved secondary metabolism including a putative efflux system and have homology to the SylA producing genes of *P. syringae*. BPSL0584-BPSL0591 encodes two hypothetical proteins (BPSL0590 and BPLS0591) with some homology to known toxins including SpvB of *Salmonella enterica* and Toxin complex components of *Photorhabdus luminescens* and may represent a novel toxin cluster. These genes neighbour phage-related elements suggesting possible horizontal acquisition. A Phospholipase D-like protein is represented (BPSS1381), flanked by hypothetical proteins and core genome genes and a hemagglutinin/hemolysin related region BPSS1720-BPSS1728 with similarity to the filamentous hemagglutinin FHA of *Bordetella pertussis*.

**Figure 4:**
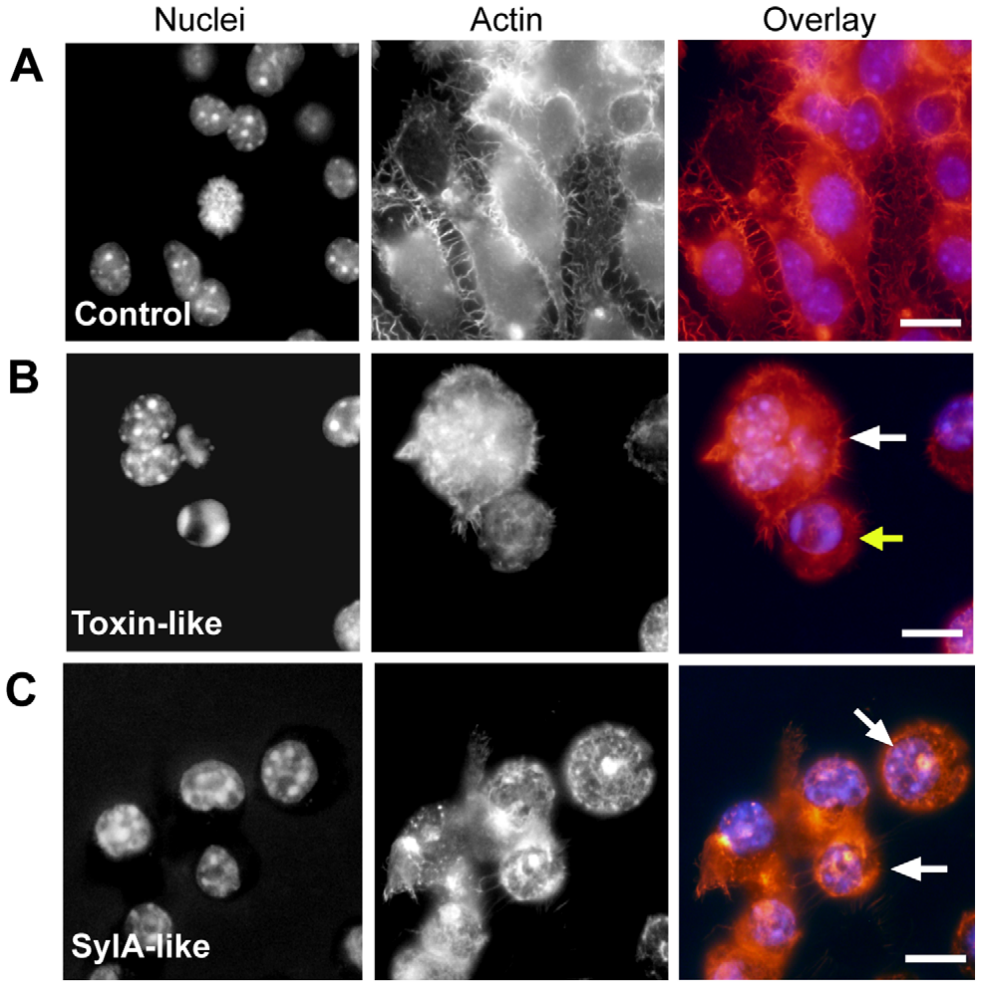
Cytotoxicity of positive clones containing regions encoding hypothetical toxin-like proteins and a SylA-like small molecule. Macrophages treated with crude lysate prepared from *E. coli* carrying vector only control (A) and those treated with clones expressing hypothetical toxin-like proteins with homology to the N-terminal of SpvB of *S. enterica* and Toxin complex components of *P. luminescens* (B) and a SylA-like component (C) for 24 h and stained with TRITC-conjugated phalloidin (red) and Hoechst 33258 (blue) to reveal cytoskeletal and nuclear structures. Macrophages treated with clones encoding the toxin- like homolog display both multi-nucleation (white arrow) and apoptosis (yellow arrow). Macrophages treated with the SylA-like (BylA) clone lysates are shrunken and display a punctate actin cytoskeleton with regions of condensation (white arrows). Scale bars  = 10 µm.

In order to begin to characterize the range of likely cellular phenotypes caused by this plethora of new candidate anti-macrophage factors we focused on the four positive clusters diagrammed in Figure 3. These clusters carry CDSs encoding factors related to secondary metabolism (NRPS/PKS), candidate enzymes, bacterial adhesins and unclassified hypothetical proteins. The NRPS/PKS encoding region BPSS1263-BPSS1269 has homology to the Syringolin A (SylA) producing NRPS cluster from the plant pathogen *Pseudomonas syringae* [25]. SylA is a proteome inhibitor and is cytotoxic to a number of mammalian cell types, including carcinoma derived cell lines, such compounds are therefore of interest as potential anticancer drugs [25], [26]. Macrophages treated with positive clones containing the SylA-like NRPS cluster show aberrations in nuclear morphology, with nuclei becoming enlarged and showing an irregular (here termed ‘corrugated’) perimeter. The cytoskeleton of the treated cells also becomes amorphous, punctate and collapses around the nucleus itself (Figure 4 C).

Thirty of the anti-macrophage loci contained ‘hypothetical proteins’ whose potential functions cannot be predicted from homology with known virulence factors. BPSL0590 and BPSL0591 are CDSs found in a positive locus on chromosome 1 which encode such hypothetical proteins. However closer examination of protein predictions from these two CDSs does reveal some limited homology to known toxins from other bacterial pathogens suggesting they may encode novel toxins. Position-specific-iterative blast (psi-BLAST) reveals that this putative membrane protein also has a central region similarity to the Rhs associated core sequence (1.98e-10) and to the middle N and C-terminal domains of a *Photorhabdus luminescens* insecticidal Toxin complex (Tc) component protein, specifically TcdB N and C terminal regions (7.87e-36 and 7.90e-29 respectively). The N-terminal region of BPSL590 has homology to the N-terminal of *Salmonella enterica* plasmid virulence associated protein SpvB (1.03e-09). The function of the N-terminus of SpvB remains unknown but shares sequence similarity to the N-terminal domain of *P. luminescens* toxin TcaC, the function of TcaC is largely unclear but is thought to act as a potentiator modifying other toxin components and it is required to reproduce full toxicity of the Toxin complexes via recombinant expression [27], [28]. The catalytic domain of SpvB responsible for ADP-ribosylation of host cell actin is located at the C-terminus. Depolymerisation of host cell actin by SpvB causes destruction of cytoskeletal structure and cell death via apoptosis [28], [29]. Structural predictions for SpvB suggest that the C-terminus is linked to the N-terminus via a poly-proline region. This suggests that it could represent a class of modular toxins in which the active/enzymic region is linked to the N-terminus whose function remains undefined.

The neighbouring hypothetical protein BPSL0591 also displays predicted homology to a *P. luminescens* insecticidal Tc protein, specifically TccB. Macrophages treated with lysate from clones encompassing these toxin-like genes show both formation of multinucleated cells and apoptotic nuclei, (Figure 4B). These results suggest that this is an exciting new genomic island encoding toxins with putative activity on the actin cytoskeleton or the small GTPases.

**Figure 5:**
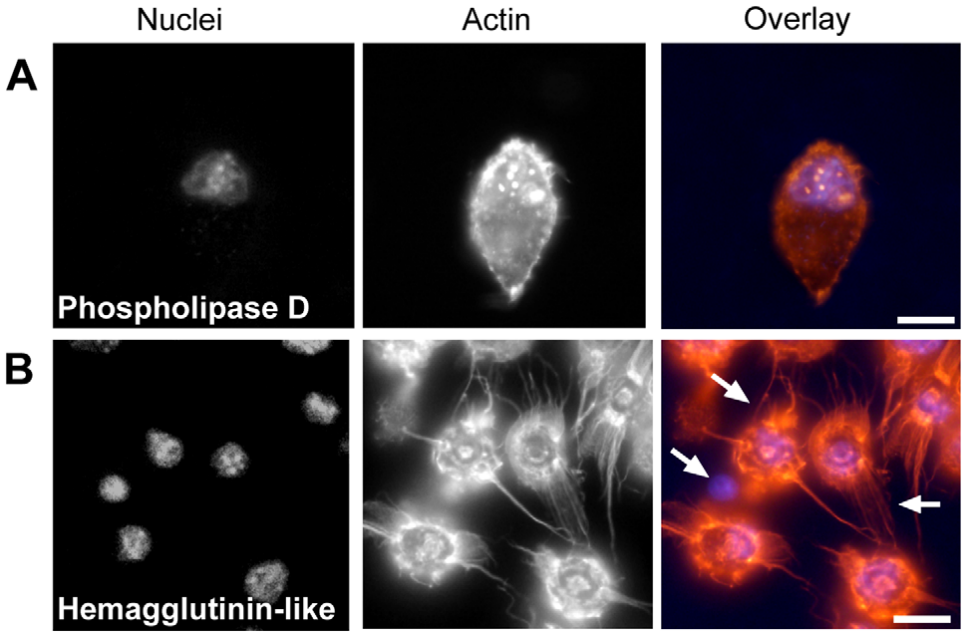
Disruption of macrophages by clones encoding a phospholipase D-like and hemagglutinin-like effector. Treatment of macrophages with lysate from clones carrying a putative phospholipase D gene for 24 h results in a loss of cytoskeletal structure and abnormalities within the nucleus (A). In contrast, macrophages treated with lysate containing a hemagglutinin-like protein for 24 h (B) suffer severe alterations in their actin cytoskeleton producing pronounced actin filaments (white arrows). Scale bars  = 10 µm.

The third cluster chosen for further phenotypic analysis (Figure 3C) carries a gene predicting a protein with putative phospholipase D activity (BPSS1381), neighbouring an endonuclease/exonuclease/phosphatase family protein (BPSS1382). This positive gene cluster also contains a putative membrane magnesium transporter protein. Macrophage monolayers treated with a preparation of clones (*E. coli* cells, whole cell lysate and supernatant) containing this region of interest show a dramatic decline in cell density, indicating a potent degree of cytotoxicity. Macrophages surviving such treatment become distended with little remaining cytoskeletal or nuclear structure (Figure 5A).

The fourth cluster chosen for follow up analysis contains two CDSs encoding a putative hemagglutinin and with homology to the large filamentous hemagglutinin precursor, FhaB, (BPSS1727) and hemolysin activator-like protein precursor, FhaC (BPSS1728) of *Bordetella pertussis* (Figure 3D). The *B. pertussis* filamentous hemagglutinin (FHA) is an adhesin and facilitates attachment to the host cell during infection following secretion which is dependent on FhaC [30]. Alongside this role in attachment, FHA is also described as having accessory functions, including pro-apoptotic activity towards macrophages [31]. Macrophages exposed to preparations of clones containing the *B. pseudomallei* hemagglutinin-like gene display a very interesting phenotype with formation of dramatic actin projections or ‘tails’ extending towards neighboring cells from shrunken cell bodies containing condensed nuclei.

### Fractionation of bioactivity from the SylA-like gene cluster

**Figure 6:**
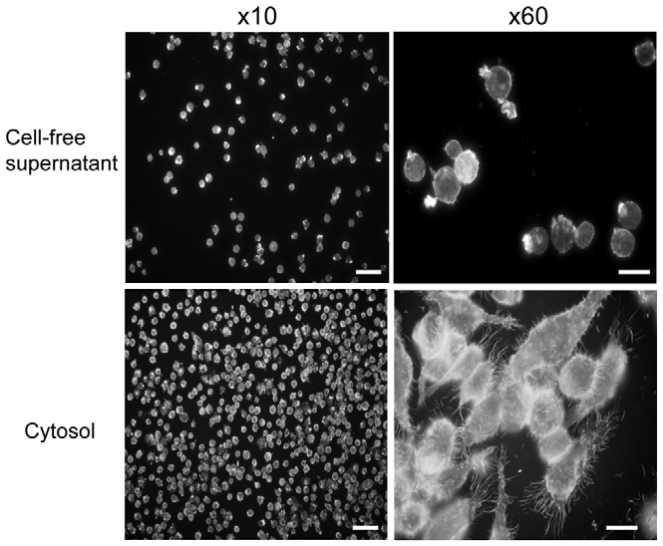
Bioactivity of clones encoding the SylA homolog is associated with the cell free supernatant. Fraction screening of clones containing the SylA-like region reveals that cytotoxicity is not present in the cytosolic fraction. F-actin cytoskeleton staining of the treated cells reveals an intact macrophage monolayer (x10) with cells displaying a normal phenotype. Cytotoxicity is found in the bacterial cell-free supernatant a clear reduction in the number of macrophages in the monolayer can be seen. Higher magnification reveals cytoskeletal collapse and cell shrinkage implying that the anti-macrophage moiety is secreted. Scale bars: x10 = 50 µm, x60 = 10 µm.

Finally, to demonstrate how bioactivities from positive gene clusters can be further confirmed and fractionated, we carried out a more detailed analysis of the gene cluster encoding the *Burkholderia* ‘SylA’ homolog, here termed BylA. The SylA gene product is a small molecule secreted into the supernatant of cultures of *P. syringae* bacteria [32]. Cell-free supernatants from recombinant *E. coli* clones carrying the *Burkholderia* BylA encoding cluster also show strong cytotoxic activity and remaining macrophages from treated monolayers display a shrunken and rounded phenotype (Figure 6). Such activity is absent from the cytosolic fraction of the same preparations showing that the bioactive component, BylA, is secreted.

## Discussion

The bacterium *B. pseudomallei* has a complex and poorly understood infection cycle involving periods in the environment and periodic infection of man. Although a serious human pathogen many of the virulence mechanisms of *B. pseudomallei* remain to be elucidated. *Burkholderia* infect and replicate within host macrophages and subsequently induce macrophage cell death but the mechanisms whereby they effect anti-macrophage activity are not completely understood. Here we have described a gain of function screen which successfully and rapidly detected over 100 anti-macrophage encoding gene clusters within genomic libraries of *B. pseudomallei* expressed in recombinant *E. coli*. This screen pulls out genomic factors either equipping *E. coli* with toxic elements which kill macrophages or an improved ability to evade them or improve growth, thus killing the cells by overwhelming/ nutrient depletion (e.g. hydrogen peroxide scavenging, drug export or biofilm formation). In this light we will now briefly discuss three aspects of the data and its generation. First, the overall distribution of the screening hits across the two chromosomes of *B. pseudomallei*. Second, the problems and pitfalls associated with the delivery of potential effectors from the recombinant *E. coli* and their uptake by the target macrophages. Third, and finally, we will spend some time discussing the likely nature of the positive clusters themselves and how they may inform our future understanding of *Burkholderia* infection biology.

### Distribution of positive gene clusters

The genomic locations of the gene clusters encoding anti-macrophage effectors and compounds are worthy of brief discussion before a detailed discussion of the clusters themselves and their potential role in infection biology. Thus although positive loci are distributed relatively uniformly across the two chromosomes of *B. pseudomallei* many are specifically associated with genomic islands and/or regions of altered GC content. Genomic islands are often linked to symbiosis or pathogenicity [16] and the co-location of anti-macrophage clusters within, or next to, GIs may confirm their suspected roles as pathogenicity islands. An alternative explanation for the association of positive loci with regions of low % GC could be that the DNA encoding them is closer to the % GC content of the host *E. coli*, and therefore these regions may be more preferentially expressed in comparison to those with high %GC.

### Secretion from recombinant *E. coli* and effector uptake by macrophages

Given the relatively crude and simple nature of the screen and given that the recombinant *E. coli* are not in any way secretion proficient or enhanced (e.g. via the addition of a T3SS), it is interesting to speculate how the anti-macrophage factors (proteins, small molecules or modified external structures such as LPS) are released from the host *E. coli* and indeed how they are taken up by (or contacted by in the context of ‘displayed’ molecules such as LPS) the target macrophages in cell culture. In this latter respect it is critical to note that the presence of some intact *E. coli* in the crude lysates applied to the macrophages appears necessary for toxicity in some cases, possibly by facilitating uptake of, or response to, recombinant factors. In contrast to much of the previous work on the three T3SSs in *Burkholderia* we would not expect T3SSs or their effectors to be recovered as positives in this system for a number of reasons. First, given that the size of the gene clusters encoding a complete T3SS can be extensive we were not expecting to recover T3SS related hits in the size range genomic fragments currently used (10–40 kb). More importantly we did not ‘arm’ the recombinant *E. coli* used in the library screening with a functional T3SS and we therefore also did not expect to identify any T3SS delivered effectors (in the absence of a clone encompassing an entire native T3SS). Finally, in relation to secretion, a number of positive clusters on chromosome 2 contain T6SS related CDSs. The precise role of T6SSs are largely uncharacterized but expression of one system has been shown to be induced upon invasion of macrophages. However mutation studies showed no alteration in invasion or intracellular survival suggesting a role in another area of pathogenesis [11]. In conclusion, and as predicted, the T3SS and T6SSs involved in invasion were not detected by this screen which focused on non-T3 and T6SSs largely by virtue of their predicted absence from the average size of cloned genome fragments.

### Cluster composition and likely effectors

In reference to the genetic composition of the clusters themselves, phenotypic analysis of four regions of interest detected by the screen begin to link activity of the gene products to some of the important phenotypes associated with *Burkholderia* infection and may allow us to begin to answer how bacterial cells persist within and spread between infected macrophages. Several of these positive gene clusters are therefore worthy of further discussion here. The first such region contains homology to the *B. pertussis* filamentous hemagglutinin or FHA. FHA is an adhesin which facilitates attachment to host cells during infection. Alongside this role in attachment, FHA also has other accessory functions, including pro-apoptotic activity towards host macrophages [31] and it is suggested that this may be used to combat the host cell-mediated immune response whilst infection is being established. Positive clones recovered in our screen, and containing a *B. pseudomallei* region homologous to FHA, cause dramatic actin protrusions from macrophages and apoptotic nuclei. It should be noted that *B. pseudomallei* travel down actin protrusions in order to spread to neighboring cells via actin based motility [12] and that the effector, BimA, has been observed as required for this activity as mutants in this gene do not induce formation of actin tails [33]. These FHA homolog induced actin protrusions may therefore play a central role in regulating the actin cytoskeleton for adherence of *B. pseudomallei* to the host cell or perhaps act as an effector, like BimA, involved in the process of intracellular spread itself.

Following attachment, *B. pseudomallei* is capable of invading and replicating within both phagocytic and epithelial cells either entering via cell mediated phagocytosis or via the combined effects of Bsa T3SS and its internally delivered effector, BopE [9]. Once inside the host cell *B. pseudomallei* cells exhibit actin based motility and induce host cell fusion, resulting in the formation of ‘multi-nucleated giant cells’ or MNGCs. Here we have described both a MNGC-like and pro-apoptotic phenotype linked to a novel toxin cluster containing genes predicting proteins with homology to the *Salmonella enterica* virulence associated protein SpvB and different components of the Toxin complexes (Tcs) of the insect pathogen *Photorhabdus luminescens*. Whilst we have not designated specific phenotypes to specific genes within this novel cluster we suggest that these genes are responsible for reorganization of the actin cytoskeleton possibly directly or via effects on the small Rho GTPases. Further, this suggests that the formation of MNGCs in *Burkholderia* infected hosts can be induced by a single factor, independently of the actin-based motility mechanism. Further work addressing the process of secretion and mechanism of action of this novel toxin cluster will be important in understanding the role this region plays in MNGC formation.

Anti-macrophage activity is also seen in response to positive clones containing a phospholipase D domain protein. A PLD gene encoding a protein with phospholipase D activity is associated with phagosomal escape in *Rickettsia prowazekii* [34]. Mutants of PLD in *Rickettsia* show attenuated virulence in guinea pigs, and animals immunized with the mutant strain are protected from subsequent challenge with the wild-type strain. Phospholipase D is also a major virulence determinant of *Corynebacterium pseudotuberculosis* and plays a key role in macrophage death [35]. Mutation of PLD genes in the pathogens *Corynebacterium pseudotuberculosis* and *Rickettsia prowazekii* have therefore shown promise as a strategy for development of attenuated strain vaccines [34], [36]. Again the role of the PLD-like gene in the interaction of *Burkholderia* with host macrophages therefore warrants further attention.

Finally, many of the positive gene clusters are associated with the production of NRPS/PKS systems which are predicted to make small molecules or peptides. Whilst it is often possible to predict the likely structure of the small molecules made via the unique combinations of PKS modules present, the role of these gene products in bacterial virulence is often less clear. We focused on one such positive region in *B. pseudomallei* which appears to encode a molecule similar to the proteome inhibitor SylA from *P. syringae*, which we term BylA here. SylA is of extreme interest as it shows good activity against carcinoma derived cell lines and BylA may therefore be similarly interesting and important. We have shown that gene clusters encoding BylA produce an active compound cytotoxic towards macrophages when expressed in recombinant *E. coli*. Moreover, this bioactivity can be localized to the supernatant of the recombinant *E. coli* culture suggesting that it does indeed correspond to a secreted small molecule similar to SylA.

### Conclusions and outlook

Current models for studying the factors involved *B. pseudomallei* virulence include gene knockout within the pathogen and subsequent testing for avirulence using *in vivo* models. However, *B. pseudomallei* has a large genome with high genomic plasticity between strains and subspecies, and is designed to survive in a diverse array of environmental settings. Different strains of *B. pseudomallei* possess very different genetic complements suggesting there may be a variety of mechanisms for producing the same disease outcome. These factors imply that functional redundancy is highly likely among the mechanics of infection and that other genes may compensate for those mutated, and therefore mask the loss of virulence associated with any single gene knock-out or knock-down. The gain-of-function screen described here allows us to look at the effects of single factors provided that their potency can be reconstituted in recombinant *E. coli*. Characterisation of such leads will allow us to understand poorly understood mechanisms behind the pathogenicity of this organism and also present us with putative targets for vaccine and drug development. For screening other bacteria, such as Gram-positives, and indeed other microbes, such as fungi, we can envisage that such gain of function screens could be conducted in alternative and more appropriate hosts such as *Bacillus* or indeed yeast. Such studies would then allow for the rapid and facile screening of other microbial genomes for virulence factors for which similar assays can be derived.

## Materials and Methods

### Genomic library construction

A combination genomic BAC and fosmid libraries, were used in these experiments. The BAC library was constructed in *E. coli* DH10B containing pBACe3.6 with an average *B. pseudomallei* DNA insert size of 20 kb originally employed for the Sanger genome sequencing project [14]. The Fosmid library was created using the CopyControl Fosmid Library Production Kit with *E. coli* EPI-300-T1^R^ (Epicentre) with an average insert size of 40 kb. BAC and fosmids libraries were arrayed into 96 well microplates to give ∼10X coverage of the genome. All clones in the libraries were then end-sequenced to facilitate location of their paired ends in the genome.

### Macrophage Toxicity Screening

Library plates were replicated into 96 well microplates containing 100 µl Luria Bertani medium plus 12.5 µg ml^−1^ chloramphenicol as the selective antibiotic for both the BAC and Fosmid library clones. Replicate library plates were grown for 24 h at 350 rpm, 37°C and cultures were subsequently harvested by centrifugation at 4,000 rpm for 10 minutes. 80 µl of supernatant aspirated the remaining bacterial pellet and supernatant mixed thoroughly with 80 µl 1 mg ml^−1^ lysozyme in Phosphate Buffered Saline solution. Plates were then incubated at room temperature for a minimum of 1 h, followed by three freeze-thaw cycles before centrifugation at 3,000 rpm for 10 minutes. 80 µl of the crude lysates were removed and applied to 96 well plates containing confluent monolayers of the BALB/c monocyte macrophage cell line J774-2 (from The European Collection of Cell Cultures, ECACC) in Dulbecco's Modified Eagles Medium (DMEM) supplemented with 10% foetal bovine serum, 5% non-essential amino-acids and 5 µg ml^−1^ chloramphenicol. Crude lysates and macrophages were co-incubated for 24 h (37°C, 5% CO_2_). Media on the macrophages was then replaced with phenol red-free DMEM containing an antibiotic cocktail: ampicllin 100 µg ml^−1^, gentamicin 50 µg ml^−1^, penicillin 100 U ml^−1^, streptomycin 100 µg ml^−1^, kanamycin 100 µg ml^−1^ and tetracycline 5 µg ml^−1^ and incubated with the macrophages for 2 h (37°C, 5% CO_2_) to destroy live bacteria which would otherwise affect the readout of the cell viability assay. Macrophage cell viability was assessed using the XTT assay [37]. Candidate positive BAC and Fosmid library clones were selected for their ability to reduce viability by 40% or more comparative to untreated cells.

### Candidate Identification

BAC and fosmid end sequences were aligned to the *Burkholderia* chromosomes using SSAHA2 version 2.5 [38] and the output exported in gff format. The alignments were manually checked to verify their integrity and to ensure the correct distance (10–20 kb) between mate-paired sequences. Once the gff had been edited it was uploaded to a custom *Burkholderia* GBrowse database, and the BAC and fosmid sequences were displayed as separate tracks in the GBrowse detail panel. This allows for the identification of ‘clusters’ of clones containing putative virulence factors. These positive ‘clusters’ form due to the multiplicity of genomic coverage within a fosmid library, for example, up to ten clones encoding an anti-macrophage toxin might be recovered from a library possessing 10X genomic coverage. The minimum region of genetic overlap within clusters was examined for candidate CDSs or operons using the annotated K96243 genome and BLASTX analysis [6]. The distribution and location of positive loci on chromosome 1 and 2 were diagrammed using DNAPlotter [39].

### Characterisation of Cellular Phenotypes

Confluent monolayers of J774-2 macrophages on glass coverslips were treated with equivalent volumes of crude lysates (prepared as described above) from clones identified as containing regions of interest and co-incubated for 24 h. Coverslips were then washed in sterile 1X PBS before fixing with 4% paraformaldehyde (w/v) in PBS for 15 min. Coverslips were then washed in 1 X PBS and immersed in a fresh solution of ammonium chloride in 1X PBS (13.3 mg/ml) for 15 minutes, at room temperature followed by washing in 1X PBS. Macrophages were permeabilised by covering with 0.2% Triton X-100 in 1X PBS for 15 minutes. Staining of the filamentous actin cytoskeleton was carried out with TRITC conjugated phalloidin (Sigma), at a 1/500 dilution in 1X PBS by inverting the coverslip onto a 60 µl drop of the staining solution and incubating at room temperature in the dark for 1 h. Following incubation the coverslips were washed 3×5 minutes in 1X PBS with the first wash containing 0.12 µg/µl^−1^ Hoechst 33258 (Sigma) to stain the nuclei. A final wash by brief immersion 2X in distilled water is then made and coverslips mounted onto slides using ProLong Gold Antifade (Molecular Probes, Invitrogen) before visualization using fluorescence microscopy. For fraction screening of the SylA homolog a single colony of a BAC library clone containing the SylA region of homology was picked and grown for 24 h in LB plus 12.5 µg ml^−1^ chloramphenicol. Bacteria were harvested by centrifugation at 7,000 rpm for 5 minutes and the culture supernatant removed. Cell-free supernatant was prepared by filter-sterilising with a 0.22 µm syringe-driven filter unit (Millipore). The cytosolic fraction was prepared by re-suspending the bacterial pellet in 1X PBS and sonicating the mixture. The resultant sonicated product was centrifuged at 10,000 rpm for 15 min. to remove cell debris from the cytosol preparation. Supernatant and cytosol fractions were applied to J774 macrophage monolayers at 1∶5 (v/v) supernatant or cytosol: culture media and co-incubated for 24 h before fixing and staining as described.

## Supporting Information

Table S1 Complete inventory of anti-macrophage associated loci identified on *B. pseudomallei* K96243 chromosome 1
(DOC)

Table S2 Complete inventory of anti-macrophage associated loci identified on *B. pseudomallei* K96243 chromosome 2
(DOC)

## References

[1] Dance DA (2002). Melioidosis.. Curr Opin Infect Dis.

[2] White NJ (2003). Melioidosis.. Lancet.

[3] Adler NR, Govan B, Cullinane M, Harper M, Adler B (2009). The molecular and cellular basis of pathogenesis in melioidosis: how does *Burkholderia pseudomallei* cause disease?. FEMS Microbiol Rev.

[4] Rotz LD, Khan AS, Lillibridge SR, Ostroff SM, Hughes JM (2002). Public health assessment of potential biological terrorism agents.. Emerg Infect Dis.

[5] Galyov EE, Brett PJ, Deshazer D Molecular Insights into *Burkholderia pseudomallei* and *Burkholderia mallei* Pathogenesis.. Annu Rev Microbiol.

[6] Stevens MP, Wood MW, Taylor LA, Monaghan P, Hawes P (2002). An Inv/Mxi-Spa-like type III protein secretion system in *Burkholderia pseudomallei* modulates intracellular behaviour of the pathogen.. Mol Microbiol.

[7] Burtnick MN, Brett PJ, Nair V, Warawa JM, Woods DE (2008). *Burkholderia pseudomallei* type III secretion system mutants exhibit delayed vacuolar escape phenotypes in RAW 264.7 murine macrophages.. Infect Immun.

[8] Muangsombut V, Suparak S, Pumirat P, Damnin S, Vattanaviboon P (2008). Inactivation of *Burkholderia pseudomallei* bsaQ results in decreased invasion efficiency and delayed escape of bacteria from endocytic vesicles.. Arch Microbiol.

[9] Stevens MP, Friebel A, Taylor LA, Wood MW, Brown PJ (2003). A *Burkholderia pseudomallei* type III secreted protein, BopE, facilitates bacterial invasion of epithelial cells and exhibits guanine nucleotide exchange factor activity.. J Bacteriol.

[10] Stevens MP, Haque A, Atkins T, Hill J, Wood MW (2004). Attenuated virulence and protective efficacy of a *Burkholderia pseudomallei* bsa type III secretion mutant in murine models of melioidosis.. Microbiology.

[11] Shalom G, Shaw JG, Thomas MS (2007). In vivo expression technology identifies a type VI secretion system locus in *Burkholderia pseudomallei* that is induced upon invasion of macrophages.. Microbiology.

[12] Kespichayawattana W, Rattanachetkul S, Wanun T, Utaisincharoen P, Sirisinha S (2000). *Burkholderia pseudomallei* induces cell fusion and actin-associated membrane protrusion: a possible mechanism for cell-to-cell spreading.. Infect Immun.

[13] Sun GW, Lu J, Pervaiz S, Cao WP, Gan YH (2005). Caspase-1 dependent macrophage death induced by *Burkholderia pseudomallei*.. Cell Microbiol.

[14] Holden MT, Titball RW, Peacock SJ, Cerdeno-Tarraga AM, Atkins T (2004). Genomic plasticity of the causative agent of melioidosis, *Burkholderia pseudomallei*.. Proc Natl Acad Sci U S A.

[15] Haase A, Janzen J, Barrett S, Currie B (1997). Toxin production by *Burkholderia pseudomallei* strains and correlation with severity of melioidosis.. J Med Microbiol.

[16] Ho Sui SJ, Fedynak A, Hsiao WW, Langille MG, Brinkman FS (2009). The association of virulence factors with genomic islands.. PLoS One.

[17] Waterfield NR, Daborn PJ, Dowling AJ, Yang G, Hares M (2003). The insecticidal toxin makes caterpillars floppy 2 (Mcf2) shows similarity to HrmA, an avirulence protein from a plant pathogen.. FEMS Microbiol Lett.

[18] Sim SH, Yu Y, Lin CH, Karuturi RK, Wuthiekanun V (2008). The core and accessory genomes of *Burkholderia pseudomallei*: implications for human melioidosis.. PLoS Pathog.

[19] Tuanyok A, Tom M, Dunbar J, Woods DE (2006). Genome-wide expression analysis of *Burkholderia pseudomallei* infection in a hamster model of acute melioidosis.. Infect Immun.

[20] Pukatzki S, McAuley SB, Miyata ST (2009). The type VI secretion system: translocation of effectors and effector-domains.. Curr Opin Microbiol.

[21] Suarez G, Sierra JC, Erova TE, Sha J, Horneman AJ (2010). A type VI secretion system effector protein, VgrG1, from *Aeromonas hydrophila* that induces host cell toxicity by ADP ribosylation of actin.. J Bacteriol.

[22] Harland DN, Dassa E, Titball RW, Brown KA, Atkins HS (2007). ATP-binding cassette systems in *Burkholderia pseudomallei* and *Burkholderia mallei*.. BMC Genomics.

[23] Matsuura M, Kawahara K, Ezaki T, Nakano M (1996). Biological activities of lipopolysaccharide of *Burkholderia (Pseudomonas) pseudomallei*.. FEMS Microbiol Lett.

[24] Arjcharoen S, Wikraiphat C, Pudla M, Limposuwan K, Woods DE (2007). Fate of a *Burkholderia pseudomallei* lipopolysaccharide mutant in the mouse macrophage cell line RAW 264.7: possible role for the O-antigenic polysaccharide moiety of lipopolysaccharide in internalization and intracellular survival.. Infect Immun.

[25] Groll M, Schellenberg B, Bachmann AS, Archer CR, Huber R (2008). A plant pathogen virulence factor inhibits the eukaryotic proteasome by a novel mechanism.. Nature.

[26] Coleman CS, Rocetes JP, Park DJ, Wallick CJ, Warn-Cramer BJ (2006). Syringolin A, a new plant elicitor from the phytopathogenic bacterium *Pseudomonas syringae* pv. syringae, inhibits the proliferation of neuroblastoma and ovarian cancer cells and induces apoptosis.. Cell Prolif.

[27] ffrench-Constant R, Waterfield N (2005). An ABC Guide to the Bacterial Toxin Complexes.. Adv Appl Microbiol.

[28] Otto H, Tezcan-Merdol D, Girisch R, Haag F, Rhen M (2000). The spvB gene-product of the *Salmonella enterica* virulence plasmid is a mono(ADP-ribosyl)transferase.. Mol Microbiol.

[29] Libby SJ, Lesnick M, Hasegawa P, Weidenhammer E, Guiney DG (2000). The *Salmonella* virulence plasmid spv genes are required for cytopathology in human monocyte-derived macrophages.. Cell Microbiol.

[30] Jacob-Dubuisson F, Buisine C, Mielcarek N, Clement E, Menozzi FD (1996). Amino-terminal maturation of the *Bordetella pertussis* filamentous haemagglutinin.. Mol Microbiol.

[31] Abramson T, Kedem H, Relman DA (2001). Proinflammatory and proapoptotic activities associated with *Bordetella pertussis* filamentous hemagglutinin.. Infect Immun.

[32] Waspi UBD, Winkler T, Ruedi P, Dudler R (1998). Syringolin, a Novel Peptide Elicitor from *Pseudomonas syringae* pv. *syringae* that Induces Resistance to *Pyricularia oryzae* in Rice'.. The American Phytopathological Society.

[33] Stevens MP, Stevens JM, Jeng RL, Taylor LA, Wood MW (2005). Identification of a bacterial factor required for actin-based motility of *Burkholderia pseudomallei*.. Mol Microbiol.

[34] Driskell LO, Yu XJ, Zhang L, Liu Y, Popov VL (2009). Directed mutagenesis of the *Rickettsia prowazekii* pld gene encoding phospholipase D.. Infect Immun.

[35] McKean SC, Davies JK, Moore RJ (2007). Expression of phospholipase D, the major virulence factor of *Corynebacterium pseudotuberculosis*, is regulated by multiple environmental factors and plays a role in macrophage death.. Microbiology.

[36] Hodgson AL, Carter K, Tachedjian M, Krywult J, Corner LA (1999). Efficacy of an ovine caseous lymphadenitis vaccine formulated using a genetically inactive form of the *Corynebacterium pseudotuberculosis* phospholipase D.. Vaccine.

[37] Scudiero DA, Shoemaker RH, Paull KD, Monks A, Tierney S (1988). Evaluation of a soluble tetrazolium/formazan assay for cell growth and drug sensitivity in culture using human and other tumor cell lines.. Cancer Res.

[38] Ning Z CA, Mullikin JC (2001). SSAHA: a fast search method for large DNA databases.. Genome Res.

[39] Carver T, Thomson N, Bleasby A, Berriman M, Parkhill J (2009). DNAPlotter: circular and linear interactive genome visualization.. Bioinformatics.

