# Supplementary material for: Genome-Wide Analysis Reveals Loci Encoding Anti-Macrophage Factors in the Human Pathogen Burkholderia pseudomallei K96243
Source: PLoS One. 2010 Dec 22;5(12):e15693. doi: 10.1371/journal.pone.0015693 (PMC3008741; doi:10.1371/journal.pone.0015693)
Supplement: Table S1 — Complete inventory of anti-macrophage associated loci identified on B. pseudomallei K96243 chromosome 1 (DOC) [file pone.0015693.s001.doc]

**Table S1 Complete inventory of anti-macrophage associated loci identified on *B. pseudomallei* K96243 chromosome 1**

| Hit # | Genetic Region (bp) | CDS coordinates | No. of clones | | Features within region |
| --- | --- | --- | --- | --- | --- |
| 1 | 28900,39500 (10.6 kb) | BPSL0027-BPSL0039 | 2 | - Flagellar biosynthetic protein  - Hypothetical protein  - Methyltransferase  - Two component regulatory system sensor kinase protein  - ABC transporter, ATP binding domain  **ABC transporter # 84 = Class III importer OTCN** | |
| 2 | 49000,62300 (13.3 kb) | BPSL0045-BPSL0056 | 3 | - type III restriction system endonuclease (17f02 and 16f04 only)  - AraC family regulatory protein  - Putative ABC transporter  - putative phenylacetaldehyde dehydrogenase  - conserved hypothetical protein  - putative short chain dehydrogenase  - glucose-methanol-choline (GMC) oxidoreductase family protein  **- ABC transporter #29 = Class III importer HAA** | |
| 3 | 148600,156200 (7.6kb) | BPSL0130-BPSL0146 | 5 | **ON GI 2**  **Repeat region**  - Hypothetical protein BPSL0130  - zinc finger CHC2-family protein  - Bacteriophage related proteins | |
| 4 | 163300,166000 (2.7 kb) | BPSL0152-BPSL0154 | 6 | **ON GI 2**  **Repeat region**  Bacteriophage tail protein | |
| 5 | 177400,182900 (5.5 kb) | BPSL0173-BPSL0177 | 4 | **ON GI 2**  **Repeat region**  Putative phage portal vertex protein | |
| 6 | 298800,315300 (16.5 kb) | BPSL0286-BPSL0298, (partial) | 2 | - metal ion transporter, metal ion (Mn2+/Fe2+)  - carbohydrate porin  - natural resistance-associated macrophage protein | |
| 7 | 332200,358800 (26.6 kb) | BPSL0313-BPSL0335 | 2 | - UDP-N-acetylglucosamine pyrophosphorylase  - outer membrane porin | |
| 8 | 475200-482200 (7.0 kb) | BPSL0436-BPSL0465 | 7 | - phosphoenolpyruvate-protein phosphotransferase  - glutamate--cysteine ligase | |
|  | 490900, 511000 (20.1 kb) | BPSL0452-BPSL0465 | 5 | - cytochrome c oxidase polypeptide I  - hypothetical protein BPSL0471  - error-prone DNA polymerase  - ABC-type transporter, periplasmic component  **ABC transporter BPSL0466 = Class III No 8 DLM BP BPSL0467-69 = NEW1 unknown function** | |
| 9 | 616000,624300 (8.3 kb) | BPSL0563-BPSL0568 | 3 | **ON GI 3**  - hypothetical protein BPSL0565  - von Willebrand factor type A  - Subtilisin-like serine protease  - phage integrase family protein | |
| 10 | 648800,664200 (15.4 kb) | BPSL0584-BPSL0591 | 2 | GI 3 OVERLAP- hypothetical protein BPSL0590, BPSL0591 - FG-GAP repeat/YD repeat/RHS repeat protein  - phage integrase family protein | |
| 11 | 705300,730300 (25 kb) | BPSL0623-BPSL0644 | 2 | oxidoreductase, FAD-binding | |
| 12 | 771400,780600 (9.2 kb) | BPSL0677-BPSL0683 | 5 | - putative asparagine synthetase B  - 3-phosphoshikimate 1-carboxyvinyltransferase  - HAD-superfamily hydrolase | |
| 13 | 817800,832600 (14.8 kb) | BPSL0716-BPSL0723 | 3 | - hypothetical protein BPSL0719  - glycosyl transferase family 51  - membrane carboxypeptidase | |
| 14 | 868100,894600 (26.5 kb) | BPSL0752-BPSL0769 | 2 | **ON GI 4**  - hypothetical protein BPSL0764  - SNF2-related:helicase  - ATP phosphoribosyltransferase  - putative phospholipase protein | |
| 15 | 910300,920600 (10.3 kb) | BPSL0783-BPSL0792 | 2 | - peptidase  - O-antigen polymerase family protein BPSL0791  - serine protease | |
| 16 | 952000,962100 (10.1 kb) | BPSL0818-BPSL0828 | 4 | - glutathione dependent alcohol dehydrogenase  - AraC family transcription regulator  - PfkB family kinase | |
| 17 | 981700,991800 (3.95 kb) | BPSL0843-BPSL0846 | 2 | - 2-hydropantoate 2-reductase  - major facilitator family transporter | |
| 18 | 1013300,1047700 (34.4 kb) | BPSL0873- BPSL0897 | 2 | - Drug resistance transporter EmrB/QacA  - translation elongation factor G  -*sodB* | |
| 19 | 1105000,1112400 (7.4 kb) | BPSL0945-BPSL0953 | 5 | **ON GI 5**  - putative type I restriction-modification methylase  - hypothetical protein  - putative replication protein | |
| 20 | 1115300,1132400 (17.1 kb) | BPSL0956- BPSL0973 | 2 | - dihydroxy acid dehydratase  - leucyl aminopeptidase | |
| 21 | 1199900,1213800 (13.9 kb) | BPSL1030-BPSL1043 | 2 | - ABC transporter, ATP-binding protein  - ABC transporter, permease protein  - putative two-component system, sensor kinase  **ABC transporters:**  **Class III Familiy OTCN #90 (BPSL1039-BPSL1040)**  **Class III Family PAO #97 (BPSL1030-BPSL1033)** | |
|  | 1218200,123570 (17.9 kb) | BPSL1048-BPSL1068 | 2 | - ABC transporter, ATP-binding protein  - hypothetical proteins  **ABC transporter Class II Family ART #2** | |
| 22 | 1268200,1289000 (20.8 kb) | BPSL1097-BPSL1110 | 2 | - DNA translocase FtsK  - cell division protein FtsK  - glycosyl hydrolase, family 15  - intracellular PHB depolymerase | |
| 23 | 1426000,1439200 (13.2 kb) | BPSL1242-BPSL1247 | 2 | metallopeptidase, M24 family BPSL1242 | |
|  | 1438600,1450100 (11.5 kb) | BPSL1247- BPSL1254 | 3 | - two-component hybrid sensor and regulator  - D-3-phosphoglycerate dehydrogenase  metallopeptidase family M24 BPSL1247 | |
|  | 1450100,1469000 (18.9 kb) | BPSL1255-BPSL1267 | 2 | - CdpA BPSL1263  - putative transport system/ multidrug resistance protein MdtB/ AcrB, AcrD, AcrF family protein (BPSL1266)  - putative transport system membrane protein (BPSL127) | |
| 24 | 1515600,1528600(13 kb) | BPSL1301-BPSL1309 | 2 | - GGDEF domain-containing protein  - 200 kDa antigen p200, putative  - ABC transporter, periplasmic substrate-binding protein  - diguanylate cyclase  - LacI family transcriptional regulator  - extracellular solute-binding protein  - non-ribosomal peptide synthetase modules and related proteins-like, putative  - hypothetical proteins  **ABC transporter BPSL1301-BPSL1303 Class III transporter, #45 Family: MOI** | |
| 25 | 1580600,1597500 (16.9 kb) | BPSL1353-BPSL1367 | 2 | - polyphosphate kinase  - FtsH endopeptidase/ATP-dependent metalloprotease FtsH BPSL1356  - phosphoglucosamine mutase | |
| 26 | 1614900,1618300 (3.4 kb) | BPSL1385-BPSL1390 | 3 | **ON GI 7**  Hypothetical proteins | |
|  | 1618900,1626000 (7.1 kb) | BPSL1392-BPSL1398 | 2 | - putative exported endonuclease BPSL1394  - outer membrane porin BPSL1397  - histone deacetylase family, putative BPSL1395 | |
| 27 | 1689000,1701700 (12.7 kb) | BPSL1452-BPSL1465 | 3 | - replicative DNA helicase  - hypothetical protein BURPS1710b_2420  - Ser/Thr protein phosphatase family protein  - family C40 unassigned family peptidase BPSL1465 | |
| 28 | 1702600,1716300 (13.7 kb) | BPSL1467-BPSL1478 | 2 | - dolichyl-phosphate-mannose-protein mannosyltransferase family protein (BPSL1474) (glycosyltransferase)  - threonine synthase  - aminotransferase AlaT  - homoserine dehydrogenase  - putative LPS biosynthesis-related protein | |
| 29 | 1717300,1730900 (13.6 kb) | BPSL1479-BPSL1492 | 2 | ClpB ATPase dependent protease, chaperonin | |
| 30 | 1791900,1800900 (18.5 kb) | BPSL1538- BPSL1551 | 3 | - MiaB-like tRNA modifying enzyme YliG  - ABC transporter, ATP-binding protein  - AsmA family protein  - aldehyde dehydrogenase (NAD) family protein  - hypothetical proteins  **ABC transporter** **BPSL1545-1546 Class III ABC transporter Family NO (unclassified) #68**  **BPSL1548 – Class II ABC transporter Familiy ART REG** | |
| 31 | 1811400,1822100 (10.7 kb) | BPSL1561-BPSL1567 | 3 | - AcrB/AcrD/AcrF family protein  -RND family multidrug efflux pump  - acriflavin reistance protein  -putative voltage-gated ClC-type chloride channel ClcB BPSL1570 (1h11 and 15f11 only, hit extends to BPSL1575) | |
| 32 | 1895300,1906400 (9.601 kb) | BPSL1634-BPSL1642 | 3 | **OVERLAPS GI 8**  - putative two-component regulatory system (BPSL1634)  - GGDEF –like protein BPSL1635  - lipase (BPSL1637)  - putative transposase  - GntR family regulatory protein (BPSL1642) | |
| 33 | 1906600,1922900 (16.3 kb) | BPSL1644-BPSL1659 | 3 | ON GI 8 - succinate-semialdehyde dehydrogenase  - outer membrane porin  - ABC transporter, ATP binding protein ABC transporter BPSL1649-BPSL1652 = Class III ABC transporter Family MOI #50 | |
| 34 | 1943800,1960800 (16.8 kb) | BPSL1667-BPSL1679 | 2 | **ON GI 8**  - hypothetical proteins  - tetratricopeptide repeat protein  - major facilitator family transporter  - putative two component system , response regulator (BPSL1669)  - outer membrane porin (BPSL1674) | |
| 35 | 1983700-1989700 (6 kb) | BPSL1705-BPSL1706 | 2 | **ON GI 8**  - tash protein pest motif family  - hemagglutinin  - Hep_Hag family  - YadA domain-containing protein  - Autotransporter adhesion  - putative HNS-like protein (BPSL1706) | |
| 36 | 1988935-2019700 (30.8 kb) | BPSL1707-BPSL1714 | 3 | - putative syringomycin synthetase/ nonribosomal peptide synthetase (BPSL1712)  - putative penicillin amidase (BPSL1710)  - carbamoyltransferase family protein (BPSL1711)  - family S45 unassigned peptidase  **BPSL1710-1714 = beginning of NRPS cluster– entire cluster not covered** | |
| 37 | 2230900-2237500 (6.6 kb) | BPSL1875-BPSL1878 | 2 | - methyl-accepting chemotaxis protein (BPSL1875)  - putative phospholipase (BPSL1876)  - phosphoesterase family protein | |
| 38 | 2634300,2647900 (13.6 kb) | BPSL2195-BPSL2204 | 2 | ABC transporter **Class III #75 family OPN** | |
| 39 | 2672100,2691000 (18.8 kb) | BPSL2228-BPSL2234 | 2 | - amino acid adenylation domain protein  - non-ribosomal peptide synthetase/ syringomycin synthetase (BPSL2229 & BPSL2232)  - major facilitator family transporter  - linear gramicidin synthetase subunit D  - multidrug efflux RND membrane fusion protein  **BPSL2214-BPSL2233 = putative NRPS cluster. Linked to putative efflux transport genes** | |
|  | 2681600,2704400 (22.8 kb) | BPSL2230-BPSL2241 | 2 | - AcrB/AcrD/AcrF family protein (BSPL2235)  - putative exported lipase (BPSL2237)  - outer membrane autotransporter  - putative non-ribosomal peptide synthase (BPSL2230-2241)  - amino acid adenylation domain protein  - acriflavin resistance protein  - RND efflux transporter  - multidrug ABC transporter (not on system inventory) | |
| 40 | 2719400,2723900 (4.5 kb) | BSPL2255-BPSL2257 | 3 | - cupin superfamily protein family  - putative lipoprotein (BPSL2257)  - metallo-beta-lactamase family protein | |
| 41 | 2774600,2788300 (13.7 kb) | BPSL2302- BPSL2309 | 4 | - Response regulator protein (BPSL2303)  - oligopeptidase A (BPSL2305)  - putative nitrite extrusion proteins (BPSL2307-2308)  - nitrate reductase, alpha subunit (BPSL2309) | |
|  | 2787600-2795400 (7.8 kb) | BPSL2310-BPSL2314 | 2 | - Putative nitrate reductase; beta, delta and gamma subunits (BPSL2310, 2311, 2312)  - putative nitrate/ nitrite sensor protein (BPSL2313)  - Putative response regulator protein (BPSL2314) | |
|  | 2795400,2821100 (26.5 kb) | BPSL2315-BPSL2334 | 3 | - endonuclease, exonuclease, phosphatase family BPSL2315  - putative nitrogen regulation protein NR (I) BPSL2316  - putative nitrogen regulation protein NR (II) BSPL2317  - glutamine synthetase ‘glnA’ BPSL2318  - putative membrane protein BPSL2321, 2322  - putative exported protein BPSL2323  - putative ATP-dependent helicase BPSL2324  - putative amino-acid acetyltransferase BPSL2325  - putative transporter protein BPSL2327  - CAIB/BAIF family protein BPSL2328  - putative acyl-CoA dehydrogenase BPSL2329  - LysR family regulatory protein BPSL2330  - hypothetical protein BPSL2319, BPSL2320, BPSL2326, BPSL2331, BPSL2332, BPSL2333, BPSL2334 | |
|  | 2818700,2821200 (1.97 kb) | BPSL2332-BPSL2334 | 3 | - hypothetical proteins  - StaB  - plasmid stabilisation system  - addiction module toxin, RelE/StbE family  - addiction module antitoxin | |
| 42 | 2838300, 2850500 (12.2 kb) | BPSL2349-BPSL2356 | 4 | - deoxyribodipyrimidine photolyase  - alkane 1-monooxygenase  -putative nitric oxide reductase | |
| 43 | 2851300,2862300 (11 kb) | BPSL2358-BPSL2367 | 4 | - U32 family peptidase (BPSL2362)  - coproporphyrinogen III oxidase (BPSL2366)  - methyl-accepting chemotaxis protein (BPSL2367)  - hypothetical proteins | |
| 44 | 3059900,3069300 (9.4 kb) | BPSL2538-BPSL2544 | 2 | - adenosine deaminase  - aminopeptidase N  - hypothetical proteins  - xanthine/uracil permease family protein | |
| 45 | 3082100,3089100 (7.0 kb) | BPSL2553- BPSL2559 | 3 | - outer membrane porin protein  - exonuclease, DNA polymerase III, epsilon subunit family/GIY-YIG catalytic domain protein  - putative siderophore receptor protein  - TonB-dependent receptor | |
| 46 | 3088900,3096300 (7.4 kb) | BPSL2560-BPSL2566 | 2 | - GTP pyrophosphokinase  - (p)ppGpp synthetase I, SpoT/RelA | |
| 47 | 3141200,3157600 (18.2 kb) | BPSL2615-BPSL2630 | 2 | **ABC transporter** **Class III family PAO #93 (BPSL2615-2617)**  **-** UbiD family decarboxylase | |
| 48 | 3172500,3175700 (3.2 kb) | BPSL2650-BPSL2652 | 3 | **ABC transporter Class III family HAA # 28 (BPSL2561 and 2562 partial)** | |
|  | 3175900,3186000 (10.1 kb) | BPSL2652-BPSL2662 | 2 | - urease subunits and accessory proteins | |
| 49 | 3200300,3206900 (6.6 kb) | BPSL2675-BPSL2679 | 3 | - wbiF – putative glycosyl transferase  - wbiE – putative glycosyl transferase  - wbiD – putative O-antigen methyl transferase  - wbiC - putative glycosyl transferase | |
|  | 3207500-3232800 (25.3 kb) | BPSL2680-BPSL2702 | 2 | - wzt ABC transporter ATP binding component  -wzm ABC transporter membrane permease  - rm1D  - rm1C  - rm1A  - dTDP-glucose 4.6-dehydratase rm1B  -putative 1-acyl-SN-glycerol-3-phosphate acytransferase plsC  - dihydroorotase-like protein pyrX  - pyrB aspartate carbamoyltransferase BPS2690  -pyrR bifunctional regulator/ uracil phosphoribosyltransferase BPSL2691  - Chaperonins groEL and groES1  - Putative kinase BPSL2696  **ABC transporter Class I ABC transporter Family CLS #7** | |
| 50 | 3372100,3384000 (11.9 kb) | BPSL2821-BPSL2830 | 2 | - molecular chaperone DnaK  - para-aminobenzoate synthase, component I **PabB** BPSL2825 | |
| 51 | 3473600,3489600 (16 kb) | BPSL2904-BPSL2921 | 2 | - tyrosyl-tRNA synthetase  - anhydro-N-acetylmuramic acid kinase | |
| 52 | 3589600,3593500 (3.0 kb) | BPSL3012-BPSL3015 | 3 | glutamate N-acetyltransferase/amino-acid acetyltransferase **argJ** | |
| 53 | 3685000,3696700 (26.9 kb) | BPSL3078-BPSL3096 | 2 | - CspD cold shock-like protein BPSL3079  - Putative DnaK-type chaperone BPSL3080  - peptidase, M1 family BPSL3089  - colicin V processing peptidase BPSL3093  - putative toxin secretion ABC transporter  - putative bacteriophage related peptidase BPSL3096  - TolC family type I secretion outer membrane protein  **ABC transporter Class I Family DPL # 11 (BPSL3092-3094)** | |
| 54 | 3708600,3714100 (16.7 kb) | BPSL3101-BPSL3117 | 2 | **ON GI 10**  - ClpB protease associated ATPase (partial) BPSL3101  - Hypothetical proteins/ membrane proteins  - type VI secretion protein (EvpB family?)  **- Repeat regions BPSL3114-3117**  **- BPSL3103 and 3110 Type VI secretion (tss-1 = BPSL3111-3097)** | |
| 55 | 3727100,3732800 (5.7 kb) | BPSL3122-BPSL3127 | 4 | - Cytochrome b/b6, N-terminal domain petB BPSL3122  - serine protease DegQ (BPSL3125)  - Sec-independent protein translocase proteins tatC and tatB | |
| 56 | 3782400-3802000 (19.6 kb) | BPSL3177-BPSL3198 | 2 | - engB putative GTP-binding cell division protein (BPSL3182)  - preprotein translocase, SecY subunit BPSL3193  - DNA-directed RNA polymerase subunit alpha | |
| 57 | 3813600,3827500 (13.9 kb) | BPSL3219-BPSL3225 | 3 | DNA-directed RNA polymerase subunit beta | |
| 58 | 3881700,3895500 (13.8 kb) | BPSL3265-BPSL3279 | 2 | **Overlaps GI 11**  - putative plasmid replication protein (BPSL3270)  - hypothetical proteins  - rare lipoprotein A family protein (rlpA BPSL3276)  - metallo-beta-lactamase family protein (BPSL3277)  - putative phospholipid-binding protein  - cation efflux family protein | |
| 59 | 3978600,4012700 (34.1 kb) | BPSL3356-BPSL3380 | 3 | - ATP-dependent DNA helicase Rep  - glycine cleavage system proteins gcvT, gcvH, gcvP  -putative heavy metal resistance membrane ATPase BPSL3378 | |
