# Supplementary material for: Genome-Wide Analysis Reveals Loci Encoding Anti-Macrophage Factors in the Human Pathogen Burkholderia pseudomallei K96243
Source: PLoS One. 2010 Dec 22;5(12):e15693. doi: 10.1371/journal.pone.0015693 (PMC3008741; doi:10.1371/journal.pone.0015693)
Supplement: Table S2 — Complete inventory of anti-macrophage associated loci identified on B. pseudomallei K96243 chromosome 2 (DOC) [file pone.0015693.s002.doc]

**Table S2 Complete inventory of anti-macrophage associated loci identified on *B. pseudomallei* K96243 chromosome 2**

| Hit  # | Genetic region (bp) | CDS coordinates | No. of clones | Features within region |
| --- | --- | --- | --- | --- |
| 1 | 1,8700 (8.7 kb) | BPSS0001-BPSS0009 | 3 | - 2-amino-3-ketobutyrate coenzyme A ligase (Kbl) BPSS0005  - phage integrase family protein |
| 2 | 49400,60600 (11.2 kb) | BPSL0051-BPSL0058 | 2 | - chromate resistance transport protein Chr A BPSS0053  - chromate resistance exported protein ChrB BPSS0054  - UvrA excinuclease ABC, A subunit BPSS0058 (partial)  **ABC transporter**  **Class III Family UVR #105** |
| 3 | 75300,81200 (5.9 kb) | BPSS0066-BPSS0070 | 3 | - non-hemolytic phospholipase C precursor (BPSS0067)  - twin-arginine translocation pathway (BPSS0066)  - integrase catalytic region |
| 4 | 87100,112400 (25.3 kb) | BPSS0077A-BPSS0092 | 5 | - hypothetical protein BPSS0078  - Rhs element Vgr protein  - cytochrome c oxidase, subunit I  - Type V secretory pathway, adhesin AidA  - PqaA protein  - Hep_Hag family protein BPSS0088  - hypothetical proteins  **Type I fimbriae BPSS0091 and BPSS0092 (partial)** |
| 5 | 116000,122000 (6.0 kb) | BPSS0096-BPSS0101 | 3 | - hypothetical protein BPSS0098  - EvpB family type VI secretion protein  - OmpA family protein  - putative cytoplasmic protein  **BPSS0095-BPSS0116 = Type VI secretion system-2** |
| 6 | 127800,133200 (5.4 kb) | BPSS0104-BPSS0105 | 3 | - hypothetical protein BPSS0104  - Rhs element Vgr protein BPSS0105 |
| 7 | 182500,191700 (9.2 kb) | BPSS0142-BPSS0145 | 2 | - putative glucan 1,4-alpha-glucosidase  - putative amylase  - ribose ABC transporter, ATP-binding protein  - ATP-dependent RNA helicase DbpA  - DEAD/DEAH box helicase domain protein  **ABCtransporter Class III Family MOS # 58 BPSL0140-0142** |
| 8 | 217300,239600 (22.3 kb) | BPSS0165-BPSS0178 | 2 | - type VI ImcF/SciS family protein BPSS0167  - OmpA family membrane protein BPSS0168  - chaperone clpB BPSS0174  - Putative lipoprotein BPSS0170  - EvpB family type VI secretion  **Type VI secretion system-3 = BPSS0185-BPSS0167** |
|  | 239700,243400 (3.7 kb) | BPSS0179-BPSS0181 | 3 | - SciB protein BPSS0179  - ImpA family type VI associated protein BPSS0180  - Rhs element Vgr protein BPSS0181 |
| 9 | 284300,299200 (14.9 kb) | BPSS0210-BPSS0221A | 3 | - radical SAM domain protein  - drug resistance transporter, EmrB/QacA family  - methyl-accepting chemotaxis protein **tar** BPSS0215  N-acylglucosamine 2-epimerase |
| 10 | 310400,316900 (6.5 kb) | BPSS0228-BPSS0232 | 4 | - hypothetical protein BPSS0229  - type I phosphodiesterase  Glyoxalase/Bleomycin resistance protein/Dioxygenase superfamily protein BPSS0230  - squalene/phytoene synthase family protein BPSS0232 |
|  | 316300,333200 (16.9 kb) | BPSS0233-BPSS0244 | 2 | - TonB-dependent hemoglobin/transferrin/lactoferrin receptor family protein  - penicillin-binding protein, 1A family BPSS0238  - putative hemin ABC transport system, ATP-binding protein BPSS0240  - putative hemin ABC transport system, membrane protein BPSS0241  -putative hemin transport system,  substrate-binding protein BPSS0242  - putative hemin ABC transport system-related protein BPSS0243  - putative exported heme receptor protein BPSS0244  **ABC transporter Class III Family ISVH #38** |
| 11 | 447200,472700 (25.5 kb) | BPSS0320-BPSS0339 | 2 | amino acid dioxygenase BPSS0339  iron-sulfur binding protein BPSS0322  amino acid transporter, putative BPSS0330  aldehyde dehydrogenase (NAD) family protein BPSS0329 |
| 12 | 532400,543600 (11.2 kb) | BPSS0386-BPSS0402 | 3 | **Overlaps GI 13**  - hypothetical proteins  - bacteriophage/transposase fusion protein |
| 13 | 562800,570400 (7.6 kb) | BPSS0410-BPSS0414 | 2 | - Hypothetical proteins BPSS0410 to 413  - acetolactate synthase |
| 14 | 617100,633800 (16.7 kb) | BPSS0452-BPSS0463 | 2 | - helix-hairpin-helix motif domain/PHP domain protein  - multicopper oxidase family protein BPSS0456  - FAD-dependent oxidoreductase  - putative copper-resistance exported protein BPSS0458  - putative copper-resistance membrane protein BPSS0459  - putative methyl-accepting chemotaxis protein BPSS0460  - hypothetical protein BPSS0463 |
|  | 634500,638100 (3.6 kb) | BPSS0464-BPSS0468 | 3 | - putrescine ABC transporter, ATP-binding protein  - putrescine ABC transporter, periplasmic putrescine-binding protein  - potH, potG, potF  **ABC transporter Class III Family MOI #52** |
| 15 | 666700,674400 (7.7 kb) | BPSS0491-BPSS0496 | 3 | - alkyl hydroperoxide reductase subunit  - chitin binding domain-containing protein |
| 16 | 717100,719600 (2.5 kb) | BPSS0525-BPSS0526 | 2 | - hypothetical protein BPSS0525 |
| 17 | 731800,750000 (18.2 kb) | BPSS0537-BPSS0548 | 3 | - putative glycosyl transferase BPSS0537  - drug resistance transporter, EmrB/QacA family protein (HylD) BPSS0541  - glycosyl hydrolase BPSS0542  - levanase  - glutathione-independent formaldehyde dehydrogenase  - transcriptional regulator, AraC family  - serine hydroxymethyltransferase |
|  | 749900,763200 (13.3 kb) | BPSS0549-BPSS0558 | 4 | - NADH:flavin oxidoreductase / NADH oxidase family protein  - iron-sulfur cluster binding protein  - hypothetical protein BURPS1710b_A2114  - amino acid permease |
| 18 | 811900,833600 (21.7 kb) | BPSS0591-BPSS0610 | 3 | - TonB-dependepnt Fe(III)-pyochelin receptor  - iron-regulated membrane protein  - siderophore transporter, RhtX/FptX family  - sigma-54 activated regulatory protein  - permease protein  - LysR family regulatory protein  aldehyde dehydrogenase (NAD) family protein  - TauD/TfdA family dioxygenase  - thiamine pyrophosphate enzyme family protein  - phosphoenolpyruvate phosphomutase  putative hydrolase |
| 19 | 853500,885000 (31.5 kb) | BPSS0626-BPSS0651 | 2 | - putative drug efflux protein BPSS0625  - phospholipase, patatin family BPSS0632  - amylo-alpha-1,6-glucosidase  - peptidase  - ‘mreB’ rod shape-determining protein BPSS0633  - glyoxalase/bleomycin resistance protein/dioxygenase superfamily protein BPSS0639  - sensor kinase protein BPSS0642  - AraC family regulatory protein BPSS0648 |
| 20 | 925100,938400 (13.3 kb) | BPSS0685-BPSS0696 | 3 | - monoxygenase  - sensor kinase protein BPSS0687  - 5-carboxymethyl-2-hydroxymuconate semialdehyde dehydrogenase |
| 21 | 1060000,1070400 (10.4 kb) | BPSS0794-BPSS0797 | 4 | - oxidoreductase, short chain dehydrogenase/reductase family  - hypothetical protein BURPS668_A1165  - hemagglutinin family protein / Hep_Hag family protein BPSS0796  - chromosome segregation ATPase  - IclR family regulatory protein |
| 22 | 1075600,1091400 (15.8 kb) | BPSS0803-BPSS0813 | 2 | - Putative hemolysin BPSS0803  - diguanylate cyclase/cyclic diguanylate phosphodiesterase  - aromatic amino acid transport protein  - aromatic amino acid aminotransferase  - hypothetical protein BPSS0812  sensor histidine kinase BPSS0813 |
|  | 1091200,1104100 (12.9 kb) | BPSS0814-BPSS0819 | 3 | - putative chemotaxis protein BPSS0814  - penicillin-binding protein 1C BPSS0816  - alpha-2-macroglobulin domain protein BPSS0817 |
|  | 1110400,1128100 (17.7 kb) | BPSS0825-BPSS0840 | 3 | - isopenicillin N epimerase BPSS0826  - microbial collagenase  - monooxygenase family protein  - universal stress family protein  - oxidoreductase, zinc-binding dehydrogenase family  **Collagenase = subfamiliy M9A unassigned peptidase with similarity to *Vibrio parahemolyticus* PrtVp** |
| 23 | 1258900,1270000 (11.1 kb) | BPSS0957-BPSS0961 | 2 | - hypothetical protein BPSS0957  - Rhs element Vgr protein BPSS0958  - Rhs-related membrane protein/YD repeat protein BPSS0960  **BPSS0958 not previously documented as a Vgr-like protein in K96243** |
| 24 | 1275000,1295000 (20 kb) | BPSS0965-BPSS0976 | 3 | - oxidoreductase alpha (molybdopterin) subunit BPSS0969  - GntR family transcriptional regulator BPSS0970  - Mg(2+) transport ATPase, P-type 2 ’mgtB’ BPSS0973  - Subfamily S8A unassigned peptidase BPSS0974 |
| 25 | 1302600,1315600 (13 kb) | BPSS0984-BPSS0995 | 2 | - UDP-N-acetylglucosamine 1-carboxyvinyltransferase, putative BPSS0984  - major facilitator family transporter  - histidine kinase BPSS0990  - Response regulator BPSS0991  - catalase ‘katB’ BPSS0993  - methyltransferase family protein  -AraC family transcriptional regulator BPSS0995 |
| 26 | 1363200,1395300 (32.1 kb) | BPSS1008-BPSS1020 | 2 | - polyketide synthase (PksK) BPSS1008  - polyketide synthase BPSS1009  - putative halogenase BPSS1010  - putative membrane transport protein BPSS1011  - putative voltage-gated clc-type chloride channel BPSS1012  - putative LysR family transcriptional regulator BPSS1015  - putative ionic antiporter BPSS1016  - putative antibiotic resistance protein BPSS1017  - putative fumarylpyruvate hydrolase BPSS1019  putative AraC family transcriptional regulator BPSS1020 |
| 27 | 1418000,1437100 (19.1 kb) | BPSS1041- BPSS1059 | 3 | **On GI 15**  **Repeat region**  - heavy metal efflux pump CzcA BPSS1041  - bacteriophage replication gene A protein (GpA) |
|  | 1418000,1442100 (24.1 kb) | BPSS1059-BPSS1065 | 2 + | **Repeat region**  Bacteriophage related proteins  (many orphan end sequence alignments) |
| 28 | 1460300,1482200 (21.9 kb) | BPSS1089-BPSS1102 | 2 | - bacteriophage late control gene D protein BPSS1089  - alanine dehydrogenase BPSS1090  - putative capsule biosynthesis protein BPSS1097  - heat-shock chaperone protein BPSS1095-BPSS1096  - cation transport ATPase protein BPSS1100  - putative methyltransferase |
| 29 | 1488000,1494800 (6.8 kb) | BPSS1110-BPSS1114 | 3 | - putative gamma-butyrobetaine,2-oxoglutarate dioxygenase BPSS1110  - hypothetical protein  - transporter, basic amino acid/polyamine antiporter BPSS1112  - Family S54 unassigned peptidase BPSS1113  - ATP-dependent metalloprotease FtsH BPSS1114 |
| 30 | 1505400,1523600 (18.2 kb) | BPSS1121-BPSS1135 | 2 | - putative GTP cyclohydrolase protein BPSS1121  - WD repeat-containing protein BPSS1122  - FAD/FMN-binding/pyridine nucleotide-disulphide oxidoreductase family protein  - putative riboflavin biosynthesis protein BPSS1125  - putative O-methyltransferase BPSS1126  - putative transmembrane transporter protein BPSS1128  - putative LuxR family transcriptional regulator BPSS1131  - putative 2,4-dienoyl-CoA reductaseBPSS1133  - PadR-like family regulatory protein BPSS1134  - putative hydroxyethylthioazole kinase BPSS1135 |
| 31 | 1695900,1703600 (7.7 kb) | BPSS1252-BPSS1257 | 3 | - hypothetical proteins  - ribonuclease E  - major facilitator transporter family protein BPSS1252 |
|  | 1704000,1722700 (18.7 kb) | BPSS1258-BPSS1267 | 4 | - putative oxidoreductase/dehydrogenase BPSS1258  - alcohol dehydrogenase, iron-containing BPSS1259  - hypothetical protein  - OmpA family membrane protein BPSS1264  - putative peptide/siderophore synthetase BPSS1266  - putative MbtH-like protein BPSS1267  -(Methyl-accepting chemotaxis protein) |
|  | 1719700,1722600 (2.9 kb) | BPSS1267-BPSS1269 | 5 | - putative MbtH-like protein BPSS1267  - putative efflux system protein BPSS1268  - non-ribosomal peptide synthase BPSS1269 (syringomycin synthetase) |
|  | 1719600,1727800 (8.2 kb) | BPSS1267-BPSS1269 | 2 | - - putative MbtH-like protein BPSS1267  - putative efflux system protein BPSS1268  - non-ribosomal peptide synthase BPSS1269 (syringomycin synthetase) |
| 32 | 1858200,1864800 (6.6 kb) | BPSS1358-BPSS1362 | 3 | - putative sensor kinase protein BPSS1358  - putative response regulator protein BPSS1359  - putative two-component sensor kinase protein BPSS1360  - glyoxylate reductase |
| 33 | 1883600,1891900 (8.3 kb) | BPSS1377- BPSS1384 | 6 | - cytochrome c oxidase BPSS1377  - putative magnesium transporter BPSS1379  - Phospholipase D domain protein BPSS1381  - endonuclease/exonuclease/phosphatase family protein BPSS1382  - rotamase BPSS1383A  - hypothetical protein |
| 34 | 1935000,1940000 (5.0 kb) | BPSS1422- BPSS1425 | 2 | - beta-hydroxylase  - glycine betaine/L-proline ABC transporter, periplasmic glycine betaine/L-proline-binding protein BPSS1423, BPSS1425  - transcriptional regulator, AraC family protein BPSS1424  **ABC transporter Class III family OTCN # 88 BPSS1423+ 1425** |
| 35 | 1981200,1994900 (13.7 kb) | BPSS1453-BPSS1462 | 4 | - iron-sulfur cluster-binding protein, rieske family/putative carboxynorspermidine decarboxylase  - hypothetical protein  - major facilitator family transporter BPSS1458  - two-component sensor kinase BPSS1460  - putative two-component response regulator BPSS1461 |
| 36 | 2015300,2035000 (19.7 kb) | BPSS1476-BPSS1492 | 4 | - hypothetical protein  - putative GntR family transcriptional regulator protein BPSS1477  - Mandelate racemase/muconate lactonizing enzyme, C-terminal domain protein BPSS1478  - putative dehydrogenase BPSS1478A  - hypothetical protein  -putative NH(3)-dependent NAD(+) synthetase BPSS1482  - putative TetR family transcriptional regulator BPSS1483  - copper-containing nitrite reductase BPSS1487  - N-acetylmuramoyl-L-alanine amidase domain-containing protein BPSS1490  - glycosyltransferase TibC BPSS1491 |
| 37 | 2110900,2127300 (16.4 kb) | BPSS1556-BPSS1566 | 2 | - metabolite:proton symporter family protein  ­- dehydrogenase, FMN-dependent family  -MarR-family transcriptional regulator BPSS1556  - alpha-ketoglutarate permease ‘kgtP’ BPSS1558  - LysR-family transcriptional regulator BPSS1559  - serine carboxypeptidase family protein BPSS1561  - serine protease, kumamolysin BPSS1562  - phosphate transporter BPSS1566 |
| 38 | 2333600,2359100 (25.5 kb) | BPSS1699-BPSS1719 | 4 | - tryptophan synthase beta chain ‘trpB’ BPSS1699  - 2-oxoacid dehydrogenase subunit E1 BPSS1711  - succinate dehydrogenase flavoprotein subunit BPSS1718  -putative AsnC-family transcriptional regulator BPSS1710  putative AraC-family transcriptional regulator BPSS1714 |
| 39 | 2359000,2367200 (8.2 kb) | BPSS1720-BPSS1726 | 2 | - putative GntR-family transcriptional regulator BPSS1721  - malate dehydrogenase BPSS1722  - putative lyase BPSS1723  - 2-methylcitrate dehydratase BPSS1725  - aconitate hydratase BPSS1726  (hemagluttinin 78c03 only BPSS1727-BPSS1728) |
| 40 | 2433500,2448100 (14.6 kb) | BPSS1774-BPSS1789 | 3 | - AMP nucleosidase BPSS1777  - homoserine kinase BPSS1779  - putative MarR-family regulatory protein BPSS1781  - organic hydroperoxide resistance protein BPSS1782  -esterase, PHB depolymerase family BPSS1784  - putative molybdenum transport-related, exported protein BPSS1786  - putative molybdenum transport-related membrane protein BPSS1787  - putative molybdenum transport-related,ATP-binding protein BPSS1788  - molybdenum transport protein BPSS1789 |
| 41 | 2467400,2484900 (17.5 kb) | BPSS1808-BPSS1826 | 4 | - acetaldehyde dehydrogenase BPSS1808  - thioesterase BPSS1809  - branched-chain amino acid aminotransferase BPSS1810  - putative non-ribosomal peptide synthesis thioesterase BPSS1812  - NRPS related protein/ phytanoyl-CoA dioxygenase (PhyH) family protein/ BarB2 BPSS1813  - NRPS related/ BarD BPSS1815  - hypothetical protein  - putative serine/threonine protein phosphatase BPSS1819  - AraC family transcriptional regulator BPSS1824  - Glycosyltransferase BPSS1825, BPSS1826 |
| 42 | 2548400,2563900 (15.5 kb) | BPSS1876-BPSS1888 | 2 | - putative sensor kinase/response regulator fusion protein BPSS1876  - aldehyde dehydrogenase NAD family protein BPSS1878  - acetolactate synthase BPSS1879 (biofilm formation)  - Na+/ H+ antiporter BPSS1880  - hypothetical protein  - Phospholipase D, putative |
| 43 | 2571200,2580500 (9.3 kb) | BPSS1896- BPSS1902 | 3 | - 2,4-dienoyl-CoA reductase (NADPH) ‘fadH’ BPSS1898  - putative AraC-family regulatory protein BPSS1899  - putative LysR-family transcriptional regulator BPSS1900, BPSS1902 |
| 44 | 2589800,2624000 (34.2 kb) | BPSS1912-BPSS1939 | 2 | - transposase for insertion sequence element IS1001 BPSS1912  - lysine-specific permease BPSS1913  - putative metallo-beta-lactamase family protein BPSS1915  - zinc-containing alcohol dehydrogenase superfamily protein BPSS1918  - F0F1 ATP synthase subunit alpha  putative methyl-accepting chemotaxis protein BPSS1927  - putative outer membrane lipoprotein BPSS1929  - putative ABC transport system, exported protein BPSS1930  -putative ABC transport system, membrane protein BPSS1931  - putative TetR-family transcriptional regulator BPSS1935  - putative outer membrane efflux protein BPSS1936  - putative ABC transport system, exported protein BPSS1937  - putative ABC transport system, ATP-binding protein BPSS1938  **ABC transporter Class III Family o228 – Function: Drug resistance #72** |
| 45 | 2629700,2638500 (8.8 kb) | BPSS1942-BPSS1952 | 2 | - Alcohol dehydrogenase BPSS1944  - ATP synthase gamma chain BPSS1945  - ATP synthase alpha chain BPSS1946  - putative ATP synthase B subunit BPSS1947  - ATP synthase C chain BPSS1948  - putative ATP synthase A chain BPSS1949  - putative ATP synthesis-related protein BPSS1951  - putative ATP synthase epsilon chain BPSS1952 |
| 46 | 2688700,2705700 (17 kb) | BPSS1988- BPSS1998 | 2 | - hypothetical protein  - peptidase, family S15 B[SS1992  - serine metalloprotease BPSS1993  - metal-related two-component system, response regulator BPSS1994  - heavy metal TCS sensor histidine kinase BPSS1995  - class D beta-lactamase BPSS1997  - putative lipoprotein |
| 47 | 2713900,2725900 (12 kb) | BPSS2009-BPSS2015 | 3 | - glucosamine--fructose-6-phosphate aminotransferase BPSS2009  - outer membrane protein  - putative inner membrane glycosyl transferase BPSS2015 |
| 48 | 2771200,2794600 (23.4 kb) | BPSS2053-BPSS2059 | 2 | **On GI 16**  - cell surface protein/ hemagluttinin/ adhesin/ hemolysin BPSS2053  - YD repeat/RHS repeat protein BPSS2054  - Rhs element Vgr protein BPSS2056  - putative ATP-binding inner membrane transport protein BPSS2058 |
| 49 | 2802200,2819100 (16.9 kb) | BPSS2067-BPSS2081 | 3 | **On GI 16**  - aldose 1-epimerase BPSS2067  - ABC transporter, ATP-binding protein BPSS2069  - ABC transporter, substrate-binding protein  - mandelate racemase / muconate lactonizing enzyme BPSS2072  - GntR family regulator protein BPSS2073  - senescence marker protein-30 BPSS2074  - transposase, IS66 familly BPSS2076  -putative DNA-binding protein BPSS2079  - hypothetical protein  - alpha-galactosidase BPSS2081  - putative ABC transporter permease BPSS2082  **ABC transporter Class III Familiy MOS # 61 BPSS2069-BPSS2071** |
| 50 | 2946500,2970900 (24.4 kb) | BPSS2179-BPSS2022 | 2 | - hypothetical proteins  - putative glycosyltransferase BPSS2182  - putative pilus assembly-related outer membrane protein BPSS2187  - putative pilus assembly-related, exported  protein BPSS2189  - putative pilus assembly-related protein BPSS2195  - type II/IV secretion system-related protein BPSS2196  - putative AsnC-family regulatory protein BPSS2199  - aromatic amino acid aminotransferase BPSS2200  - porin related, membrane protein BPSS2202  **Putative *tad-*type pilus (complete)** |
| 51 | 3032400,3049000 (16.6 kb) | BPSS2257- BPSS2269 | 2 | - Fusion protein, ATP-binding transmembrane ABC transporter and regulatory protein BPSS2259  - phosphatidylserine decarboxylase BPSS2261  - 4'-phosphopantetheinyl transferase superfamily protein BPSS2266  **ABC transporter Class I Familiy DPL # 15**  **BPSS2259** |
| 52 | 3069700,3098800 (29.1 kb) | BPSS2283- BPSS2302 | 4 | - Sulfate transporter family protein BPSS2285  - HSP20/alpha crystallin family protein BPSS2288  - Putative porin protein BPSS2289  - AMP-binding enzyme BPSS2290  - GerE family regulatory protein BPSS2291  - taurine catabolism dioxygenase TauD, TfdA family protein BPSS2294  - putative transport protein BPSS2296  -molybdopterin oxidoreductase family protein BPSS2299  - iron-sulfur cluster protein BPSS2300 |
| 53 | 3125100,3150900 (25.8 kb) | BPSS2323-BPSS2334 | 2 | - hypothetical protein  - Putative permease BPSS2324  - ABC-transporter ATP binding protein BPSS2325  - beta keto-acyl synthase / PKS BPSS2328  - RND efflux transporter  - putative acyl transferase BPSS2329  - putative aminotransferase BPSS2333  **Probable PKS/NRPS cluster** |
| 54 | 3158500,3166100 (7.6 kb) | BPSS2342-BPSS2348 | 3 | - GGDEF response regulator protein BPSS2342  - sensor histidine kinase/response regulator BPSS2345, BPSS2344  - NADPH-dependent FMN reductase domain protein  - putative arsenate reductase BPSS2348 |
